# Neural control of chronic stress adaptation

**DOI:** 10.3389/fnbeh.2013.00061

**Published:** 2013-08-08

**Authors:** James P. Herman

**Affiliations:** Department of Psychiatry and Behavioral Neuroscience, Metabolic Diseases Institute, University of CincinnatiCincinnati, OH, USA

**Keywords:** glucocorticoid receptor, hypothalamo-pituitary-adrenal axis, limbic system, stress-related diseases, stress habituation, stress sensitization

## Abstract

Stress initiates adaptive processes that allow the organism to physiologically cope with prolonged or intermittent exposure to real or perceived threats. A major component of this response is repeated activation of glucocorticoid secretion by the hypothalamo-pituitary-adrenocortical (HPA) axis, which promotes redistribution of energy in a wide range of organ systems, including the brain. Prolonged or cumulative increases in glucocorticoid secretion can reduce benefits afforded by enhanced stress reactivity and eventually become maladaptive. The long-term impact of stress is kept in check by the process of habituation, which reduces HPA axis responses upon repeated exposure to homotypic stressors and likely limits deleterious actions of prolonged glucocorticoid secretion. Habituation is regulated by limbic stress-regulatory sites, and is at least in part glucocorticoid feedback-dependent. Chronic stress also sensitizes reactivity to new stimuli. While sensitization may be important in maintaining response flexibility in response to new threats, it may also add to the cumulative impact of glucocorticoids on the brain and body. Finally, unpredictable or severe stress exposure may cause long-term and lasting dysregulation of the HPA axis, likely due to altered limbic control of stress effector pathways. Stress-related disorders, such as depression and PTSD, are accompanied by glucocorticoid imbalances and structural/ functional alterations in limbic circuits that resemble those seen following chronic stress, suggesting that inappropriate processing of stressful information may be part of the pathological process.

## The problem of chronic stress

The organismal response to stress (defined here as a real or perceived threat to homeostasis or well-being) promotes survival via adjustments to ongoing physiological processes and behavior. The activation of multiple interacting processes, including the behavioral, autonomic, endocrine, and immune systems, produces an integrated stress response. While initially adaptive, prolonged activation of molecular pathways engaged by these systems can cause pronounced changes in physiology and behavior that have long-term deleterious implications for survival and well-being. In essence, prolonged or chronic stress changes the rules under which the body regulates homeostasis, requiring new strategies for successful adaptation. This concept lies at the heart of Selye's initial description of the “general adaptation syndrome,” where after an initial “alarm” stress reaction, the organism is able to successfully manage prolonged stress for substantial periods. Only when homeostatic pressure becomes too great does the individual enter into a state of frank distress, with attendant morbidity and mortality (Selye, 1950). Importantly, the process of adaptation comes at a cost to the organism, as stress effector systems are chronically mobilized to meet the homeostatic demands of prolonged stress. Thus, stress alters the physiological milieu in a long-term manner (adaptation through change, or “allostasis”) (Sterling and Eyer, 1988), and the body's response to these changes lies at the center of both successful stress resilience as well as its transition to pathology.

The literature is replete with examples of the impact of stress on physiologic systems and behavior. For example, our group finds that exposure to a prolonged, unpredictable and non-habituating stress regimen (which we call “chronic variable stress,” or CVS) causes marked increases in cumulative glucocorticoid secretion, sensitization of hypothalamo-pituitary-adrenocortical (HPA) responses to new stressors, decreased heart rate variability, reduced weight gain, decreased sucrose preference and increased immobility in the forced swim test (Herman et al., 1995; Ulrich-Lai et al., 2006, 2007; Jankord et al., 2011; Flak et al., 2012), suggesting functional changes across a variety of neurobehavioral systems in the brain. Whereas it can be argued that the net result of these physical and behavioral changes would be maladaptive, one has to interpret them with respect to the new context of the individual. Most responses to chronic stress are adaptive, that is, beneficial to the survival of the animal. For example, increases in corticosteroid levels promote mobilization of energy, important in times of need. Corticosteroids also inhibit systems that channel resources to functions such as growth and reproduction, not necessarily of value in the midst of physiologic challenge. Similarly, behavioral changes seen during chronic stress, while at first blush “pathological,” can be argued to be beneficial in the context of chronic challenge. For example, post-stress reductions in risk assessment/behavioral withdrawal seen in so-called “anxiety” tests (e.g., elevated plus maze) (e.g., see Chiba et al., 2012) minimize risk during periods of energetic challenge. Minimizing risk may also be linked to the switch to “habitual” behaviors observed in chronically stress rodents (e.g., see Dias-Ferreira et al., 2009; Harris et al., 2012). In a hostile environment, it may make sense to exhibit behavioral withdrawal in order to reduce threat (i.e., make oneself less available for predation). Even observed changes in immobility in the forced swim test, commonly linked to depressive phenotypes, may be interpreted as a means of conserving resources during challenging times (see Hawkins et al., 1978). Thus, while responses to chronic stress can clearly have negative consequences, one has to consider the possibility that they may solve pressing problems of the organism at the expense of future success.

Chronic stress responses represent attempts at adaptation, but as noted above can create constitute physiologic challenges in and of themselves. Excess glucocorticoid secretion can impair numerous bodily systems if extended in time; enhanced sympathetic drive can lead to cardiovascular disease; and “conservative” behavioral strategies can lessen opportunities to find new food and water sources, more secure environmental surroundings and mates. Thus, at some point, the initially “adaptive” characteristics of chronic stress reactivity can cross over to the realm of “maladaptation,” defined as biological and behavioral responses that are counterproductive to the best interests of the organism. The switch from “adaptive” to “maladaptive” stress responses will be heavily dependent on the constitution of the individual, based on genetic and acquired strategies to maximize efficiency and limit overdrive of stress systems. Stress “pathologies” can arise as a result of maladaptive chronic stress responses, either as a result of systemic diseases of stress regulatory systems or pervasive activation of stress effectors in inappropriate contexts. It is important to note that the concept of “maladaptation” is not synonymous with physiologic distress. Maladaptation *per se* need not be sufficient to cause frank morbidity on its own, but may degrade the well-being of the individual or make it more vulnerable to subsequent physiologic insults.

The current review will address the problem of chronic stress, adaptation and maladaptation from the perspective of the HPA axis, perhaps the most thoroughly studied system linked to stress responses. The consequences of HPA axis activation are far-reaching, likely due to the ubiquity of glucocorticoid hormone receptors across multiple body compartments and the widespread impact of glucocorticoid hormones on gene expression. Glucocorticoid secretion is generally linked to stressful events. Consequently, glucocorticoids are often referred to as “stress hormones,” a designation that undermines appreciation of their primary functions, including redistribution of energy. Indeed, so-called “stress levels” of glucocorticoid secretion can even be observed at the peak of the circadian corticosteroid rhythm, representing a flux in hormone aimed at increasing energy supplies for the active, waking hours. With this caveat in mind, consistent activation of this system constitutes both a mechanism of stress adaptation and a potential challenge for the organism, the balance of which determines resistance or susceptibility to long-term pathologies.

## Stress adaptation

**Figure 1:**
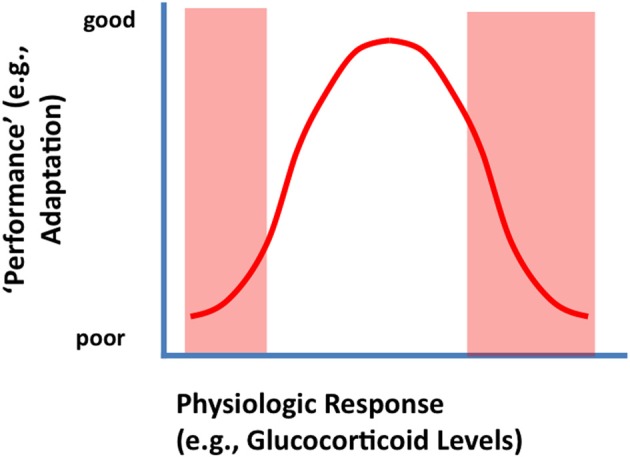
**Inverted U-shaped relationship between physiologic or behavioral “performance' and HPA axis output (corticosterone secretion).** Secretion of glucocorticoids following stress is likely an adaptive function, supplying needed energy to meet real or potential threats. Underactive stress axis activation does not mobilize the resources needed to meet a challenge, resulting in suboptimal performance (left arm of the U-shaped curve). Excessive glucocorticoid secretion (right arm of the U-shaped curve) can cause excessive or prolonged catabolic responses, which can result in turn-off of vital stress counter-regulatory systems or energetic challenge in the CNS (see text).

To be clear, glucocorticoid responses are required for survival and adaptation. The relationship between glucocorticoid secretion and adaptation (e.g., in terms of appropriate behavioral performance) is often described as an “inverted-U” shaped curve, wherein an optimal level of glucocorticoid signaling is required to produce the most effective organismal response (De Kloet et al., 1998) (Figure 1). Thus, both hypo- and hyper-secretion generate poor responses, whereas an intermediate level of corticosteroids fosters superior performance. Work from De Kloet and colleagues suggests that the molecular basis of this curious phenomenon lies in the differing binding affinities and signaling characteristics of the two primary corticosteroid receptors in brain (De Kloet et al., 1998). The mineralocorticoid receptor (MR) binds low levels of glucocorticoids, and fosters cellular activation (hippocampus) and maintains basal circadian corticosteroid rhythms. The glucocorticoid receptor (GR) binds glucocorticoids across the circadian peak/stress range, and appears to inhibit hippocampal neurons and controls the magnitude of HPA axis responses to stress via negative feedback mechanisms (Reul and Dekloet, 1985; De Kloet et al., 1998). While MR and GR share virtually identical DNA binding domains, their transactivation domains are distinct, meaning that they can have very different gene targets (Datson et al., 2001, 2008). Moreover, there is evidence that the two receptors heterodimerize (Trapp et al., 1994; Nishi et al., 2004), a process that introduces the capacity to temper specific MR and GR genomic signals and perhaps introduce new types of genomic interactions.

The right arm of the inverted U-shaped curve is likely due to potential catabolic effects of glucocorticoids on physiological and cellular functions, most likely mediated by the GR. In keeping with their role in energy redistribution, glucocorticoids promote energy mobilization, including glycogenolysis, lipolysis, and proteolysis (Munck et al., 1984). Thus, these hormones promote processes that, while good for the organism in moderation, can cause long-term cellular energy depletion at high levels. In addition to effects on catabolism, glucocorticoids also inhibit processes related to growth and reproduction (Munck et al., 1984) (the organism does not need to be concerned about growing if energy reserves are being depleted). In brain, high levels of glucocorticoids can inhibit glial glucose transport (Virgin et al., 1991) and impair neuronal survival under conditions of energetic challenge (Tombaugh et al., 1992). Glucocorticoids can also inhibit expression of key neurotrophic molecules, such as brain-derived neurotrophic factor (Smith et al., 1995; Schaaf et al., 2000), and impair hippocampal neurogenesis (Gould and Tanapat, 1999). Finally, glucocorticoids down-regulate GR expression in limbic regions controlling negative feedback (e.g., hippocampus, prefrontal c) (Mizoguchi et al., 2003; Chiba et al., 2012), which limits the ability of the system to control glucocorticoid homeostasis.

**Figure 2:**
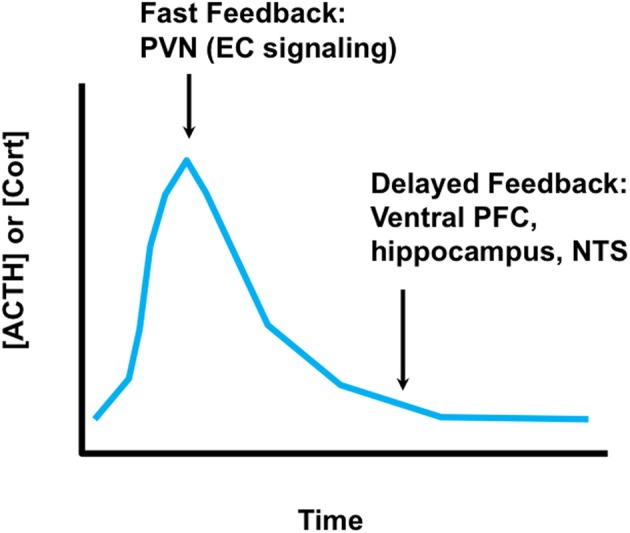
**Time domains of glucocorticoid feedback.** Fast glucocorticoid feedback occurs in the realm of minutes to seconds, and likely involves membrane effects of glucocorticoids on endocannabinoid synthesis at the PVN. Delayed feedback controls return of glucocorticoid levels to baseline, and likely involves glucocorticoid feedback signals at multiple brain regions, including the prefrontal cortex, ventral subiculum (hippocampus) and possibly the nucleus of the solitary tract. It is not clear whether delayed feedback is genomic, non-genomic or a combination of both.

Activation of glucocorticoid secretion is mediated by neuronal signals. The neuroendocrine cascade culminating in corticosteroid release is initiated by stimuli impinging on hypophysiotrophic neurons in the medial parvocellular division of the hypothalamic paraventricular nucleus (PVN). These neurons synthesize ACTH secretagogues [the most prominent of which are corticotropin-releasing hormone (CRH) and arginine vasopression (AVP)] that are released into the hypophysial portal circulation (median eminence) and transported to the anterior pituitary gland. Corticotropes respond to CRH and AVP by releasing ACTH, which is released into the systemic circulation and causes synthesis and release of glucocorticoids at the level of the adrenal gland. Glucocorticoid secretion is self-limited, undergoing end-product feedback inhibition via binding GRs in multiple brain regions as well as the pituitary gland (Keller-Wood and Dallman, 1984; Myers et al., 2012b). The net result is an HPA axis “stress response” that has a rapid onset and more gradual wane, with glucocorticoid secretion generally peaking in 15–30 min and lasting up to several hours, depending on the severity of the stressor (Figure 2). The shape of the response underscores its adaptive value in the short-term, and limits the possible negative effects of a prolonged glucocorticoid response.

Stimulation of the HPA axis occurs in reaction to or in anticipation of stress (Herman et al., 2003; Ulrich-Lai and Herman, 2009). Physiological threats (systemic stressors) initiate largely reflexive responses that can be triggered without conscious perception. However, anticipation of threat requires the organism to interpret the significance of multi-modal sensory information with respect to previous experience. Thus, stimuli that predict adversity (psychogenic stressors) can generate an HPA axis response in the absence of an existing physiologic insult. The relevance of the anticipatory glucocorticoid response hinges on the predicted need for adaptive hormonal secretion in order to redistribute resources (e.g., energy) to meet the challenge (Herman et al., 2003; Ulrich-Lai and Herman, 2009). Appropriate activation of the HPA axis by acute stress is critical, as inappropriately low reactivity can hinder physiological resilience and cognitive processes (e.g., learning and memory) (Diamond et al., 1992; Reber et al., 2007). However, many of the effects of glucocorticoids that are beneficial for short-term survival can be counterproductive or even deleterious if prolonged. Therefore, the activation and inhibition of glucocorticoid release is a temporally regulated process involving rapid neuronal activation and efficient inhibition.

Control of glucocorticoid secretion is accomplished by multiple mechanisms. The first line of defense is rapid shut-off of ACTH release by glucocorticoids secreted in response to stress. Rapid inhibition is almost certainly non-genomic, and is mediated in part by direct feedback onto PVN CRH neurons and at the pituitary (John et al., 2004; Tasker and Herman, 2011). At the PVN, so-called “fast feedback” is mediated by membrane actions of glucocorticoids, which cause local mobilization of endocannabinoids and subsequent inhibition of excitatory afferent input (Di et al., 2003; Evanson et al., 2010). Fast feedback inhibits ACTH release within minutes, and affects the amplitude of the stress response (Keller-Wood and Dallman, 1984). Glucocorticoids also act via the MR to rapidly enhance the excitability of hippocampal and basolateral amygdala neurons via a non-genomic mechanism. Both regions are upstream of the PVN, and thus binding via the MR may impact HPA drive via synaptic mechanisms (Pasricha et al., 2010). The eventual shut-off of the HPA axis stress response (return to baseline) is thought to be mediated by feedback working through limbic circuitry (Jacobson and Sapolsky, 1991; Sapolsky et al., 1991; Boyle et al., 2005; Furay et al., 2008). Shut-off occurs in the time realm of genomic actions, and may reflect cellular changes in pathways impinging on the PVN. However, it remains possible that delayed shut-off may be mediated at least in part by non-genomic signaling.

Intracellular GR (and MR) signaling is subject to modulation by other proteins, which may affect function in the context of stress. For example, the GR-binding factor FK506 binding protein 51 (also known as FKBP5) reduces glucocorticoid binding affinity and GR nuclear translocation (Binder, 2009). In PFC, chronic stress enhances expression of FKBP5, which is correlated with impaired GR nuclear trafficking and reduced expression of GR-regulated genes (Guidotti et al., 2012). Stress-related changes in expression of FKBP5 (as well as other GR binding proteins) may represent a mechanism for reduced feedback efficacy and prolonged HPA axis responses (Mizoguchi et al., 2003).

Chronic stress causes a marked reorganization of the central components of the HPA axis. Numerous studies document increases in CRH and AVP expression in parvocellular PVN neurons (Herman et al., 1995; Makino et al., 1995), consistent with increased response capacity of the central limb of the HPA axis. Chronic stress also causes marked structural plasticity in CRH neurons. Glutamatergic and NE terminal appositions on CRH somata and dendrites increase with chronic stress, consistent with enhanced excitatory drive (Flak et al., 2009). There is also evidence that PVN GABAergic signaling is impaired following chronic stress. Chronic variable stress exposure causes decreases in GABA-A receptor subunit mRNAs, which would be predicted to diminish the potential for inhibition of the HPA axis (Cullinan, 2000). At the synaptic level, chronic stress decreases miniature inhibitory post-synaptic potentials in PVN neurons (Verkuyl et al., 2004), consistent with decreased inhibitory innervation. In addition, recent studies suggest that stress causes a reversal of the cellular chloride gradient in parvocellular PVN neurons (Hewitt et al., 2009), essentially negating the inhibitory impact of GABA on post-synaptic neurons. Neuroplastic responses likely reflect increased demand upon the CRH neurons, serving to maintain response capacity if confronted with additional stressors.

Hyperactivity of the PVN may be linked to alterations in glucocorticoid feedback in brain. Numerous studies indicate that chronic stress decreases expression of GR in the prefrontal cortex (PFC) and hippocampus (Mizoguchi et al., 2003; Chiba et al., 2012). Lesion and stimulation studies indicate that both of these regions play a key role in inhibition of HPA axis stress responses, working by way of excitation of inhibitory relays into the PVN [e.g., in the bed nucleus of the stria terminalis (BST), dorsomedial hypothalamus and peri-PVN regions, among others] (Herman et al., 2003; Ulrich-Lai and Herman, 2009). Local administration of glucocorticoids into the PFC reduce HPA axis responses to stress (Diorio et al., 1993), also consistent with an important role in feedback regulation. This conclusion is further supported by studies demonstrating that forebrain deletion of the GR (including the PFC and hippocampus) (Boyle et al., 2005; Furay et al., 2008) or local knock-down of GR in the PFC (McKlveen et al., 2013) enhance HPA axis stress responses. Thus, chronic stress-induced reductions in PFC and hippocampal GR may remove an important brake on the HPA axis, resulting in down-stream changes in PVN excitability.

It is important to note that glucocorticoids also affect behavioral processes that modulate the impact of stress on the organism. Behavioral analyses indicate that glucocorticoids cause changes in learning strategy, wherein mice switch from spatial learning to stimulus-response learning in the context of acute stress or corticosterone. The switch in learning strategy is blocked by an MR antagonist, suggesting effects mediated by the MR (Schwabe et al., 2010). In addition, mice overexpressing MR fail to extinguish conditioned fear, consistent with impaired behavioral flexibility (Harris et al., 2012). Animals with forebrain deletion of GR or knockdown of GR in the PFC have increased immobility in the forced swim test (Boyle et al., 2005; McKlveen et al., 2013), supporting a role for GR in behavioral strategy selection in this context.

In contrast to the PFC and hippocampus, amygdalar structures appear to be positively regulated by chronic stress. Expression of CRH in the central nucleus of the amygdala (CeA) is increased under conditions of chronic stress or glucocorticoid excess (Makino et al., 1994, 1999; Shepard et al., 2000, 2003). Moreover, glucocorticoid implants in the CeA increase corticosterone responses to acute stress, suggesting a positive glucocorticoid feedback effect mediated by this region (Shepard et al., 2003). The CeA is linked to excitation of the HPA axis, mediated by inhibition of inhibitory relay neurons innervating the PVN (including the BST and dorsomedial hypothalamus, which are also implicated in inhibition by the PFC and hippocampus) (Herman et al., 2003). Thus, in addition to impairing feedback inhibition, chronic stress may permit feed-forward activation of the PVN by way of the amygdala.

The impact of chronic stress is also evident at the pituitary and adrenal. Chronic stress exposure causes up-regulation of proopiomelanocortin mRNA expression and protein content (Shiomi et al., 1986), consistent with enhanced capacity for release of ACTH. At the adrenal, chronic variable stress causes cellular hypertrophy and hyperplasia in the zona fasciculata of the adrenal cortex, which causes elevated responsiveness to ACTH (Ulrich-Lai et al., 2006). The PVN, pituitary and adrenal changes occur with the context of relatively small changes in resting glucocorticoid secretion, consistent with modulation of the overall capacity of the HPA axis to respond (rather than a pronounced and prolonged basal hypersecretion). The peripheral changes likely reflect the overall cumulative impact (severity) of the stress regimen, as mild or habituating regimens may not be sufficient to cause frank changes at the brain, pituitary, and adrenal level [e.g., attenuated stress-induced adrenal hypertrophy (Flak et al., 2012) and deceased induction of PVN vasopressin mRNA expression (Gray et al., 2010)].

Neurocircuit mechanisms underlying generation of chronic stress-induced HPA hyperdrive remain to be determined. In general, lesions of brain regions known to be involved in inhibition or excitation of acute stress reactivity do not affect the development of HPA-relevant chronic stress symptoms. For example, lesions of the ventral subiculum exacerbate responses to acute stress, but do not affect basal glucocorticoid secretion, adrenal hypertrophy or thymic atrophy following chronic stress (Herman and Mueller, 2006). Moreover, lesions of the medial and central amygdala, putative stress excitatory regions, do not attenuate chronic stress responses (Prewitt and Herman, 1997; Solomon et al., 2010). Thus, it is not clear that regions mediating acute stress responses are required for development of chronic stress-related HPA axis dysfunction.

## Habituation to repeated stress exposure

**Figure 3:**
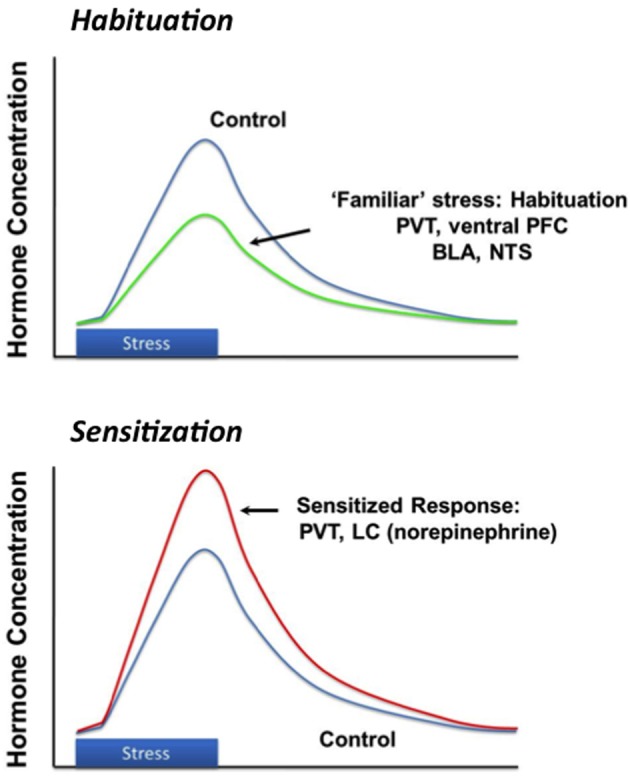
**Stress habituation and facilitation.** Repeated exposure to the same stressor results in progressive diminution of response magnitude, thought to be mediated by structures such as the prefrontal cortex (PFC) and paraventricular thalamus (PVT). Exposure to a new stressor after either homotypic or hetertypic stressors causes a larger than normal (“sensitized' or “facilitated' response), which may be mediated by enhanced drive from the basolateral amygdala (BLA), PVT or locus coeruleus (LC).

Successful adaptation to chronic stress is a dynamic process that is dependent on the attributes of the stress exposure, such as severity, modality and duration. Stress “habituation” is thought to be an important adaptive response to repeated challenge, wherein responses to a given stressor decrease upon repeated exposure and thus reduce the overall physiological burden (e.g., cumulative effects of glucocorticoid secretion) with time. Animals can generally habituate to repeated stressors (Grissom and Bhatnagar, 2009). This is evident by a marked reduction in HPA axis activation with repeated exposure to the same stimulus (Figure 3). Habituation is observed after exposure to a wide array of stimuli, ranging from mild (e.g., novel environment) to severe (limb and head immobilization) (Campmany et al., 1996; Grissom and Bhatnagar, 2009). The rate of habituation is dependent on the severity of the stressor (Garcia et al., 2000). Habituation is likely mediated by diminution of central responses to the stressor; for example, activation of c-fos expression in the medial parvocellular PVN is markedly reduced in the PVN following repeated restraint stress (Girotti et al., 2006). Habituation limits the overall physiological impact of stress, in terms of effects of repeated stress on energy balance (i.e., minimal weight loss), adrenal function (i.e., no adrenal hypertrophy) and cumulative corticosterone exposure (small decrements in thymus weight, associated with small episodic increases in corticosterone exposure) (Flak et al., 2012).

Changes in cumulative glucocorticoid exposure may be in itself a factor in the habituation process. The addition of a stress response atop the normal circadian rhythm adds to the “total” level of glucocorticoids seen by the organism, which may modulate ongoing excitability of key regulatory sites and thereby limit responsiveness. Habituation to repeated stress is blocked by pre-stress injections of an MR antagonist (Cole et al., 2000), suggesting that the process may be regulated in part by glucocorticoid signaling via the MR. Unlike variable or “severe” stress regimens [e.g., chronic social stress, chronic unpredictable stress (Chao et al., 1993; Herman et al., 1995)] repeated restraint stress does not down-regulate GR and MR mRNA expression (hippocampus) (Girotti et al., 2006), suggesting that neural feedback mechanisms remain intact. While an elevated feedback signal may be relevant to habituation, it does not constrain the HPA response to new stressors, which can be as great or greater than responses seen in stress-naïve animals (see below) (Akana et al., 1992; Marti et al., 1994).

The CNS mechanisms regulating stress habituation appear to involve limbic forebrain circuitry. Functional studies indicate that local inactivation of the ventral PFC region prior to restraint stress blocks the development of habituation to subsequent exposure, suggesting activation is necessary for the process (Weinberg et al., 2010). The basolateral amygdala also appears essential for HPA axis habituation, as local blockade of beta-adrenergic receptors after daily stress exposure attenuates reductions in ACTH and corticosterone release observed following repeated restraint (Grissom and Bhatnagar, 2011). It is important to note that the ventral prefrontal cortex has rich connections with basolateral amygdala (McDonald et al., 1999; Vertes, 2004), and thus it is possible that the two work in concert to promote habituation. Other limbic forebrain regions may also play a role in the habituation process; for example, diminished HPA axis responses to repeated noise exposure is associated with a significant increase in c-fos mRNA activation in the orbitofrontal cortex, in contrast to decreases seen in other regions of the frontal cortex (including the PFC) (Campeau et al., 2002). Like other limbic cortices, the orbitofrontal cortex has potential polysynaptic connections with subcortical limbic stress effector pathways (Price, 2007), and increased engagement may play a role in dampening stress responses.

There is also evidence for control of habituation at the level of the limbic thalamus. Work from Bhatnagar and colleagues implicate the paraventricular thalamus (PVT) in habituation of the HPA axis stress response. The PVT is a midline thalamic nucleus that interconnects with several limbic stress-regulatory regions, including the medial PFC, basolateral amygdala and bed nucleus of the stria terminalis. Lesions of the PVT block habituation of HPA axis responses to repeated restraint (Bhatnagar et al., 2002). Moreover, PVT lesions reduced the efficacy of dexamethasone feedback actions on stress-induced HPA activation following repeated but not acute restraint exposure (Jaferi et al., 2003), suggesting that effects on habituation may be linked to glucocorticoid signaling. In support of this hypothesis, local blockade of GR and MR in the PVT prevent habituation without affecting responses to acute stress (Jaferi and Bhatnagar, 2006), consistent with glucocorticoid feedback effects mediated by this region.

The habituation process may involve generalized reductions in activation of sensory signaling pathways. For example, repeated exposure causes marked reduction in restraint induced c-fos mRNA activation of primary sensory cortices and thalamic sensory relays (in addition to the PVN and limbic stress circuits), suggesting that habituation may be in part due to reduced strength or salience of perceived sensory cues (Girotti et al., 2006).

It is also possible that stress habituation is mediated by structural alterations along stress integrative circuits. For example, repeated brief restraint, a treatment that generally causes habituation, causes retraction of basal dendrites of prefrontal cortical neurons (Brown et al., 2005), which may impact down-stream regulation of HPA axis responses. More prolonged restraint-exposure paradigms (6 h/day, 21 days) produces dendritic retraction and spine loss in the PFC (Vyas et al., 2002; Cook and Wellman, 2004; Radley et al., 2006, 2008) as well as retraction in subfield CA3 of the hippocampus (Magarinos and McEwen, 1995), while causing increased dendritic complexity in the basolateral amygdala (Vyas et al., 2002). Chronic restraint also increases branching of GABAergic interneurons in the PFC (Gilabert-Juan et al., 2012), suggestive of enhanced inhibition. Structural alterations may affect the excitability of neurons, which can then alter the overall balance of limbic inputs to neurons controlling stress responsiveness.

It is important to note that habituation is not limited to repeated stressors. Chronic unpredictable or variable stress regimens also decrease physiological responses over time, although not to the same extent as homotypic regimens. For example, numerous studies indicate that the impact of chronic variable stress on body weight is most profound during the initial 1–3 days of exposure, with values plateauing thereafter (Tamashiro et al., 2007). In addition, our group has shown that chronic variable stress (CVS)-induced corticosterone hypersecretion is significantly reduced from the first to the second week of exposure (unpublished observations). Adrenal hypertrophy and thymic atrophy plateau between 1 and 2 weeks of CVS, suggesting that the impact of chronic unpredictable stress (Paskitti et al., 2000), at least in terms of the HPA axis, is not progressive.

## Chronic stress sensitization

It should be emphasized that the “stress habituated” state does not reflect return to normal physiologic status. Habituating regimens (e.g., repeated brief restraint) result in long-term changes in CNS stress circuits, including up-regulation of CRH expression in the PVN, even in the context of reduced corticosterone responses to individual restraint sessions. As noted above, despite habituation to homotypic stressors, novel stressors will induce a disproportionately large HPA axis stress response relative to acutely stressed controls (Figure 3) (Akana et al., 1992; Marti et al., 1994). Increases in CRH gene expression and enhanced HPA axis responding indicate that the underlying sensitivity of the HPA axis is increased, even though the response to the repetitive stimulus is diminished.

The PVT may mediate sensitization (facilitation) of responses to novel stressors in animals habituated to a homotypic stressor. The PVT is one of a handful of brain regions showing enhanced Fos induction following repeated stressor exposure (e.g., cold) (Bhatnagar and Dallman, 1998). As was the case with habituation, sensitization is blocked by lesions of the PVT, suggesting an important role for this region in registering stressor chronicity (Bhatnagar and Dallman, 1998). Control of HPA axis sensitization by the PVT appears to be mediated by neuropeptidergic circuits. Cholecystokinin (CCK) appears to be released in the PVT during the process of sensitization and is important in limiting the magnitude of sensitization (Bhatnagar et al., 2000). Conversely, PVT activation via the orexin pathway is required for full elaboration of stress sensitization (Heydendael et al., 2011).

Sensitization also involves noradrenergic neurons of the locus coeruleus (LC) and/or nucleus of the solitary tract (NTS). In cortex, repeated stress exposure sensitizes norepinephrine (NE) release following novel stressors (Nisenbaum and Abercrombie, 1993). Moreover, increased PVN responsiveness to NE is observed following chronic cold exposure, and appears to be required for HPA axis sensitization (Pardon et al., 2003; Ma and Morilak, 2005). Repeated homotypic stress also increases expression of tyrosine hydroxylase expression (rate limiting enzyme in NE synthesis) in the LC (Angulo et al., 1991; Mamalaki et al., 1992; Melia et al., 1992), suggesting that increased biosynthetic capacity in limbic forebrain-projecting norepinephrine neurons may play a role in the sensitization process.

Sensitization is also characteristic of models that minimize habituation, such as CVS. ACTH and corticosterone responses to novel stressors are augmented in CVS animals (e.g., see Ulrich-Lai et al., 2007). As noted, habituating and non-habituation stress regimens differ with respect to baseline endpoints (body weight, resting corticosterone, adrenal weight and/or thymus weight), but data comparing effects on the magnitude of sensitization are lacking. Thus, it is not known whether or not unpredictable regimens elicit more profound sensitization of the HPA axis.

Unpredictable stress regimens may also be relevant for understanding the lasting impact on the individual. To address this issue, we tested the long-term impact of a CVS regimen on neuroendocrine as well as behavioral outcomes. Our data indicate that CVS induces a late-emerging and long-lasting HPA axis hyporesponsivity to novel stressors (Ostrander et al., 2006), as well as impaired extinction of fear conditioning and enhanced freezing responses to reminder cues (McGuire et al., 2010). These findings indicate that chronic unpredictable stress exposure may impair resilience to future stressful experiences.

## Successful adaptation vs. “pathology”

Both habituating and non-habituating stress regimens present a challenge to the organism, and both cause sensitization of stress responses. A critical difference between the two is the cumulative impact on the body and brain. In habituating stress regimens, the ability to reduce stress axis activation over time suggests that the glucocorticoid burden may not be sufficient to cause maladaptative physiological or behavioral consequences, or that it will either take longer for cumulative damage to occur.

Maintained or enhanced stress responses to non-habituating regimens are likely linked to both severity and predictability. As noted above, stress regimens that vary in intensity habituate at different rates (Garcia et al., 2000). One could posit that a stress regimen of sufficient intensity may be able to completely block the habituation process and lead to maladaptive consequences. In addition, regimens that vary stressors or have uncertain outcomes (e.g., social stressors) may not sufficiently engage habituation mechanisms, thus allowing physiological or behavioral responses to persist for extended periods of time. It is likely that both factors are involved in determining the net impact of chronic stress exposure on the individual.

Mechanisms underlying the transition from adaptation to pathology are poorly understood. Part of the problem in defining this progression lies in determining when responses meant to be adaptive “cross over” into the realm of maladaptation. For example, at what point does elevated glucocorticoid secretion start to take a toll on the brain and body? The answer likely lies at the level of the individual, and is dependent on numerous processes including hormone clearance, MR and GR expression levels, interactions of bound receptors with nuclear co-activators and co-repressors, genetic predispositions and epigenetic modifications of receptor targets. From a neural perspective, the progression from “adaptive” to “maladaptive” is likely dependent on the degree to which the brain engages physiological responses in an appropriate context. It is appropriate to mount a glucocorticoid response in response to an imminent threat; however, engaging the HPA axis chronically or in response to innocuous cues is not.

Importantly, context plays a role in dictating how tissues respond to glucocorticoids. Equivalent levels of glucocorticoids can exert different effects on cellular function depending on stress history. For example, in hippocampus, the same dose of corticosterone down-regulates mTOR expression in chronically stressed animals, but not controls (Polman et al., 2012). mTOR is an important cell signaling pathway that is involved in neuroplasticity and subsequent control of mood (Li et al., 2010). Thus, the function of the mTOR pathway will be markedly different in the context of stress, which subsequently affects behavioral and physiological responses. These data suggest that glucocorticoids may have exaggerated impact when an organism is exposed to stress, even if absolute levels of hormone are not elevated to so-called “pathophysiologic” levels.

Animal studies tracking the adaptation/pathology transition are difficult to design, due to the lack of accompanying self-reports signifying emotional or physical discomfort characteristic of human stress-related disorders. We have attempted to address this issue by comparing neural activity in habituating (repeated restraint) vs. non-habituating (CVS) models, as the latter exhibits a more severe HPA axis “phenotype,” in terms of baseline corticosterone secretion, adrenal hypertrophy and thymic involution (Flak et al., 2012). The CVS regimen also induces behavioral changes suggestive of altered cognition (increased immobility in the forced swim test) and hedonic processing (decreased sucrose preference) (Ulrich-Lai et al., 2007; Jankord et al., 2011). Using FosB staining as a marker of long-term activation, we demonstrated that both repeated restraint and CVS procedures increase the number of activated neurons in key stress regulatory areas, including the ventral medial prefrontal cortex and the dorsomedial hypothalamus, the latter a structure linked to integration of endocrine, autonomic and behavioral stress responses. However, exposure to CVS caused FosB activation in regions not affected by repeated restraint, including the posterior hypothalamus and the NTS, both of which project to the PVN. Moreover, CVS-induced FosB expression in the medial PFC was increased significantly beyond that seen following homotypic stress exposure. In contrast, unpredictable stress did not result in decreased activation of any structures showing FosB induction by repeated restraint, suggesting that regions are not “de-recruited” (Flak et al., 2012). Thus, it is evident that unpredictable stress recruits regions not engaged by the habituating regimen, consistent with usage of distinct circuits under the two conditions.

**Figure 4:**
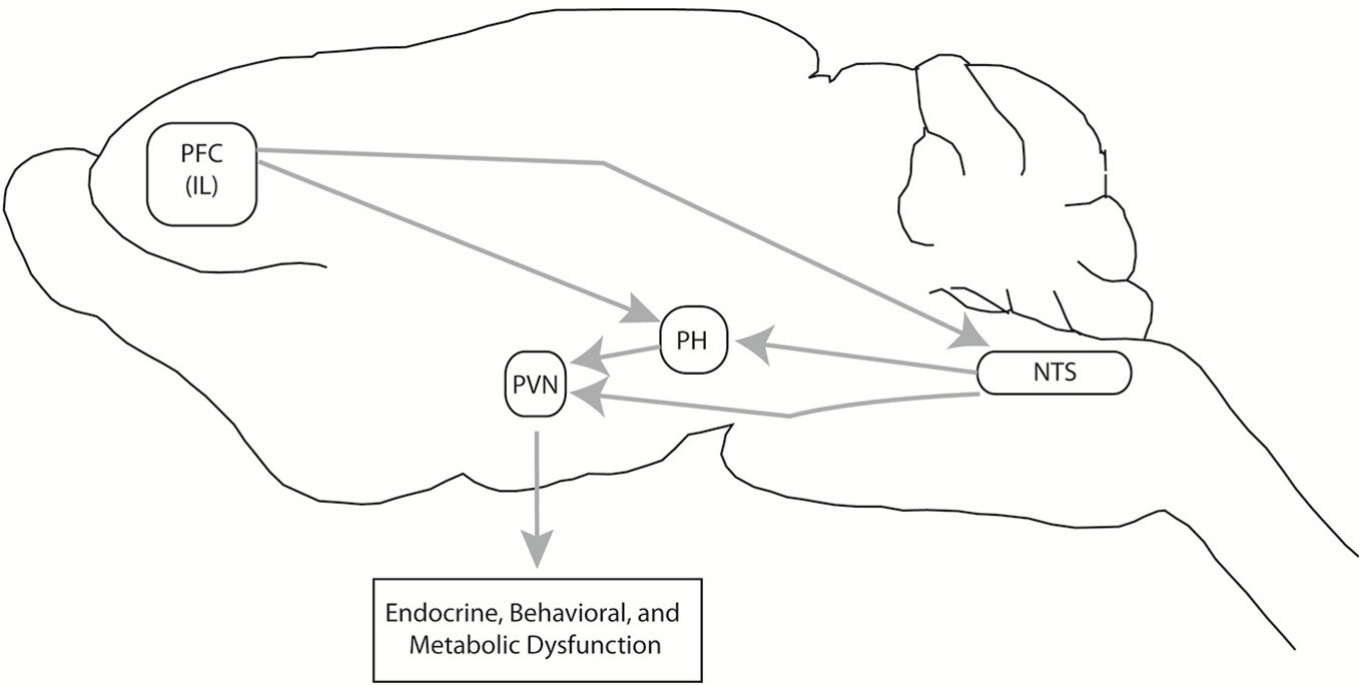
**Chronic unpredictable stress-recruited circuitry.** Exposure to chronic variable stress (CVS) selectively recruits chronic cellular activation (in terms of FosB expression) in a discrete set of interconnected brain regions, including the prefrontal cortex (PFC), nucleus of the solitary tract (NTS), and the posterior hypothalamic nucleus (PH). The PH projects to both the PH and NTS, and both PH and NTS project to the PVN, creating the opportunity for PFC-PVN regulation in parallel, in series, or both. Via this pathway, chronic stress can increase drive to the PFC and subsequently cause glucocorticoid-mediated endocrine, behavioral, and metabolic dysfunction. This figure is reprinted from Flak et al. (2012), with permission.

The regions “recruited” by chronic unpredictable stress are of substantial importance in integration of stress responses across multiple modalities. The posterior hypothalamus is involved in coordinating defensive behaviors and autonomic responses to stressors (Shekhar and Dimicco, 1987; Lisa et al., 1989). The PH also sends excitatory projections to the parvocellular PVN (Ulrich-Lai et al., 2011), and recent data from our group suggests that activation of the PH potentiates HPA axis responses to stress (Myers et al., 2012a). The NTS is traditionally thought of as an autonomic regulatory region, but also participates in HPA axis activation by both catecholaminergic and non-catecholaminergic neurons (Ulrich-Lai and Herman, 2009), and appears to play a role in regulation of anxiety-related behaviors (via GLP-1) (Kinzig et al., 2003). Both regions receive afferents from the ventral PFC (Vertes, 2004), and may together may comprise a neural circuit responsible for perpetuation of stress responses in the face of prolonged severe or unpredictable stress (Figure 4).

The enhanced HPA axis drive seen in chronic unpredictable models is likely driven by uncontrollability and uncertainty regarding outcomes. Maier's work elegantly demonstrates that controllability over a stressor (shock) can protect against the development of helplessness behavior, social inhibition and behavioral withdrawal seen following inescapable stress (Maier and Watkins, 2010). Importantly, brain regions controlling development of helplessness overlap with those recruited during exposure to unpredictable stress (i.e., ventral divisions of the medial PFC) (Maier and Watkins, 2010). Thus, continued drive of the HPA axis may be mediated by engagement of the same (or parallel) circuits that control behavioral responses to unpredictability.

Numerous psychiatric disorders are associated with dysregulation of stress responses. A substantial subpopulation of depressed individuals exhibit glucocorticoid dyshomeostasis, manifest primarily as disrupted cortisol rhythms and resistance to negative feedback (Sachar et al., 1973; Carroll, 1982; Wong et al., 2000). In fact, depression is associated with long-term HPA axis activation, manifest as adrenal hypertrophy (Amsterdam et al., 1987, 1989) as well as somatic changes indicative of increased glucocorticoid burden [e.g., accelerated bone loss (Gold and Chrousos, 2002)]. It is postulated that depression-related hypercortisolemia causes glucocorticoid “resistance” by down-regulating GRs, thereby “freeing” the HPA axis from feedback control of down-stream stress effectors (Pariante, 2004). Accordingly, depression may be an example of “out of context” corticosteroid secretion, wherein hormone release is not aligned with a true threat and is increased out of proportion with the actual objective impact of the stressor (relative to non-depressed individuals). Thus, to some extent, depression shares attributes with what may be considered a disorder of chronic stress regulation.

Conversely, PTSD is linked to low cortisol levels, which appears to be a heritable trait (Yehuda, 2009; Radley et al., 2011). In this case, enhanced glucocorticoid feedback may impair cortisol responses that are essential for normal processing of stressful information: in effect, patients are on the “left arm” of the inverted U-shaped curve relating hormone action and performance. A role for low “trait” cortisol in PTSD is supported by recent studies showing efficacy of exogenous corticosteroids in reducing symptoms in the clinic (Suris et al., 2010).

Chronic stress also causes pathological problems that extend beyond the realm of affective disorders. For example, there is a wealth of data linking chronic stress to age-related neurodegeneration and cognitive decline, likely mediated by glucocorticoid hypersecretion (see Lupien et al., 1999; Landfield et al., 2007). Chronic stress is also linked to somatic pathologies, including cardiovascular disease (see Steptoe and Kivimaki, 2012) and the metabolic syndrome (see Tamashiro et al., 2011), among others. Indeed, accumulating evidence suggests that stress has broad impact on virtually all physiological systems, with glucocorticoids serving as a contextual signal that is superimposed atop specific cellular processes.

Perhaps the key to understanding stress pathology lies in consideration of context. In depression, for example, physiological and behavioral symptoms, e.g., helplessness, anhedonia, HPA axis dysfunction and cardiovascular pathology, all mimic those induced by chronic stress regimens in animal models. The key difference between the two is the context in which these responses occur: as argued above, these behaviors and physical reactions may be entirely appropriate when an animal (or person) is confronted with environmental or physical adversity. In human depressives, the link with “actual” stress is less evident, raising the possibility that processes underlying the disorder essentially permit stress related behaviors and physical reactions to occur in the absence of threat. In depressed patients, the prefrontal cortex and amygdala, two critical components of stress circuitry, show abnormal activation patterns without any clear threat present (Drevets, 2000; Mayberg et al., 2005), suggesting the potential for engagement of responses out of context. Mounting situationally inappropriate stress responses may in turn contribute to cumulative damage associated with maladaptive aspects of stress responses (e.g., excess glucocorticoid action).

## Perspective

Considerable progress has been made in understanding neural circuits and processes responsible for chronic stress habituation, sensitization and pathology. However, the neural trigger that differentiates successful from unsuccessful coping remains elusive. Uncovering processes underlying the transition from adaptation and pathology bears consideration of what constitutes “adaptation” and “pathology,” as many responses and behaviors that may appear “maladaptive” make perfect sense in the appropriate context. Indeed, context-inappropriate physiological and emotional responses are hallmarks of stress-related disorders (depression, PTSD). Moving forward, animal models will need to incorporate an appreciation of the relevance of physiologic and behavioral endpoints to the normal repertoire of the organism under conditions of challenge, and develop testing conditions that can more clearly query how chronic stress can generate situationally inappropriate responses reminiscent of human pathology.

### Conflict of interest statement

The author declares that the research was conducted in the absence of any commercial or financial relationships that could be construed as a potential conflict of interest.
